# Risk Factors for Vaginal Colonization and Relationship between Bacterial Vaginal Colonization and In-Hospital Outcomes in Women with Obstructed Labor in a Ugandan Regional Referral Hospital

**DOI:** 10.1155/2018/6579139

**Published:** 2018-09-20

**Authors:** Joseph Ngonzi, Lisa M. Bebell, Joel Bazira, Yarine Fajardo, Dan Nyehangane, Yap Boum, Deborah Nanjebe, Adeline Boatin, Jerome Kabakyenga, Yves Jacquemyn, Jean-Pierre Van Geertruyden, Laura E. Riley

**Affiliations:** ^1^Mbarara University of Science and Technology, Department of Obstetrics and Gynecology, Mbarara, Uganda; ^2^Global Health Institute, University of Antwerp, Antwerp, Belgium; ^3^Division of Infectious Diseases, Massachusetts General Hospital, Boston, MA, USA; ^4^Massachusetts General Hospital Center for Global Health, Boston, MA, USA; ^5^Mbarara University of Science and Technology, Department of Microbiology, Mbarara, Uganda; ^6^Epicentre Mbarara Research Base, Mbarara, Uganda; ^7^Division of Obstetrics and Gynecology, Massachusetts General Hospital, Boston, MA, USA; ^8^Mbarara University of Science and Technology, Institute of Maternal Newborn and Child Health, Mbarara, Uganda

## Abstract

**Introduction:**

The proportion of women with severe maternal morbidity from obstructed labor is between 2 and 12% in resource-limited settings. Maternal vaginal colonization with group B streptococcus (GBS), *Escherichia coli*, and *Enterococcus* spp. is associated with maternal and neonatal morbidity. It is unknown if vaginal colonization with these organisms in obstructed labor women is associated with poor outcomes.

**Objectives:**

To determine whether vaginal colonization with GBS, *E. coli*, or *Enterococcus* is associated with increased morbidity among women with obstructed labor and to determine the risk factors for colonization and antibiotic susceptibility patterns.

**Methods:**

We screened all women presenting in labor to Uganda's Mbarara Regional Referral Hospital maternity ward from April to October 2015 for obstructed labor. Those meeting criteria had vaginal swabs collected prior to Cesarean delivery and surgical antibiotic prophylaxis. Swabs were inoculated onto sterile media for routine bacterial culture and antimicrobial susceptibility testing.

**Results:**

Overall, 2,168 women were screened and 276 (13%) women met criteria for obstructed labor. Vaginal swabs were collected from 272 women (99%), and 170 (64%) were colonized with a potential pathogen: 49% with *E. coli*, 5% with GBS, and 8% with *Enterococcus*. There was no difference in maternal and fetal clinical outcomes between those colonized and not colonized. The number of hours in labor was a significant independent risk factor for vaginal colonization (aOR 1.02, 95% CI 1.00–1.03, *P*=0.04). Overall, 38% of GBS was resistant to penicillin; 61% of *E. coli* was resistant to ampicillin, 4% to gentamicin, and 5% to ceftriaxone and cefepime. All enterococci were ampicillin and vancomycin susceptible.

**Conclusion:**

There was no difference in maternal or neonatal morbidity between women with vaginal colonization with *E. coli*, GBS, and *Enterococcus* and those who were not colonized. Duration of labor was associated with increased risk of vaginal colonization in women with obstructed labor.

## 1. Background

Obstructed labor is diagnosed in women presenting with features of prolonged or complicated labor, such as labor duration >24 hours, distended bladder, Bandl's ring in the lower uterine segment, fetal distress or death, edematous vulva or cervix, fetal caput or significant skull molding, and foul smelling vaginal discharge or amniotic fluid [1]. In resource-limited settings, 2–12% of women suffer severe obstetric morbidity from obstructed labor. This may include maternal sepsis, uterine rupture, postpartum endometritis, or maternal death, whereas perinatal morbidity may include neonatal sepsis, encephalopathy, or fetal or neonatal demise [2–5].

Several bacterial pathogens such as group B *Streptococcus* (*Streptococcus agalactiae*, GBS), *Enterococcus* spp., and *Escherichia coli* are known to be associated with poor obstetric and neonatal outcomes, even in women with uncomplicated labor [6–9]. We hypothesized that vaginal colonization with one or more of these known bacterial pathogens (GBS, *E. coli*, or Enterococcus spp.) is associated with poor maternal and newborn outcomes in women with obstructed labor versus those who are not colonized with those pathogens. We therefore set out to determine risk factors for bacterial vaginal colonization with a potential pathogen and the relationship between colonization and in-hospital outcomes among women with obstructed labor.

## 2. Methods

### 2.1. Study Site and Design

All women presenting to Mbarara Regional Referral Hospital (MRRH) maternity ward for delivery or postpartum care were enrolled in a larger prospective cohort study of postpartum infection at MRRH [10, 11] from which a nested substudy of women with obstructed labor were recruited between April and October 2015. MRRH is a teaching hospital for Mbarara University of Science and Technology (MUST) with 10,000 deliveries annually and a catchment of nine million people, who are largely rural dwelling. The proportion of women who were referred to MRRH maternity ward from another clinic or hospital is approximately 15%.

### 2.2. Definition of Obstructed Labor

For this study, we defined obstructed labor as the presence of two or more major features of obstructed labor (Bandl's ring, edematous vulva, edematous cervix, significant fetal skull molding, or caput), or one major plus one or more minor features of obstructed labor (foul smelling vaginal discharge, fetal distress or demise during labor, >24 hours in labor) based on published studies [1, 3]. This definition was applied uniformly to women presenting to MRRH in labor by a single obstetrician to prospectively recruit the subpopulation of women with obstructed labor.

### 2.3. Participant Enrollment and Data Collection

All women presenting to the MRRH maternity ward for delivery were approached for inclusion. Study procedures for the larger cohort included measurement of vital signs after delivery for all women; symptom questionnaire and chart review for a target subpopulation of women; and a structured physical exam, malaria testing, and blood and urine cultures for febrile and hypothermic women [10]. Among mothers consenting for the larger cohort, those meeting criteria for obstructed labor were consented for additional procedures including vaginal swab collection and structured physical exam. Those <18 years of age were enrolled as emancipated minors based on pregnancy status. Potential participants were excluded if they did not understand English or Runyankole (the local dialect) or were incapacitated and their next-of-kin declined participation. All participants provided written informed consent. Participants not previously tested for human immunodeficiency virus (HIV) within the last six months were offered HIV testing and results were recorded, given to the ward clinical care team, and shared with the participant.

After delivery, obstructed labor substudy participants were followed by a team of research nurses who administered a questionnaire which captured their sociodemographic, medical, and obstetric profiles. An abdominal examination was performed on all enrolled women 72 hours after delivery to evaluate for endometritis. Investigator-designed questionnaire responses and laboratory results were entered into a Research Electronic Data Capture (REDCap) database [12]. The final decision about delivery mode for each participant was made by the obstetric care team without investigator influence, but all enrolled women went on to deliver by Cesarean. Women were followed up at two and six weeks postpartum by phone call and interviewed about their health and the baby's health.

The current routine practice at MRRH for women in labor is to clean the vulva using chlorhexidine prior to digital vaginal examination and to give women undergoing Cesarean delivery a single dose of preoperative antibiotics (usually one gram of intravenous ampicillin) within 30 minutes of skin incision. After Cesarean delivery, women are routinely treated with combination intravenous ceftriaxone, gentamicin, and metronidazole for three days, followed by five days of oral cefixime and oral metronidazole.

### 2.4. Vaginal Swab Collection

Sterile cotton-tipped swabs were used to collect a midvaginal sample from patients meeting criteria for obstructed labor prior to Cesarean delivery and surgical antibiotic prophylaxis but after vaginal cleansing using chlorhexidine. Swabs were collected in duplicate and delivered to the Epicentre Mbarara Research Centre laboratory for analysis within one hour after collection. The first swab was used to prepare a Gram stain. The second swab was systematically inoculated onto four types of enriched and selective media and incubated at 35–37°C in ∼5% carbon dioxide for up to 48 hours, observing for growth after overnight incubation. Bacterial colonies were identified using standard biochemical methods and their identity was confirmed using the analytical profile index system (bioMérieux, Marcy-l'Étoile, France). Antimicrobial susceptibility was determined using Kirby-Bauer disk diffusion. Antibiotic breakpoints were defined using European Committee on Antimicrobial Susceptibility Testing (EUCAST) guidelines, version 5.0.

### 2.5. Outcome Variables

The primary study outcome was postpartum endometritis (puerperal sepsis) at three days postpartum, defined as infection of the female genital tract in which two or more of the following were present: pelvic pain, fever >38.0°C, abnormal vaginal discharge, and delay in the rate of reduction of the size of the uterus <2 cm/day [5]. The microbiology of puerperal sepsis cases was not evaluated due to the known polymicrobial nature of these infections and local resource constraints. Secondary study outcomes included other maternal outcomes (uterine rupture, postpartum hemorrhage, maternal death, and reoperation) and perinatal outcomes (neonatal Apgar score <7, stillbirth, and early neonatal death). The primary exposure variable was vaginal colonization with GBS, *E. coli*, *Enterococcus*, or a combination of those pathogens. Secondary exposure variables were as follows: duration of labor, HIV serostatus, duration of rupture of membranes, age, distance of residence from hospital, marital status, educational status, referral status, number of vaginal exams prior to delivery, and parity.

### 2.6. Sample Size

We calculated the sample size needed to detect a doubling of risk of postpartum endometritis from 10% to 20% between women colonized with a potential pathogen and those not colonized as 276 total participants with 80% power and an alpha error rate of 0.05.

### 2.7. Statistical Analysis

We calculated the prevalence of obstructed labor among all laboring women screened, and the proportion of women colonized with a vaginal pathogen, among those with obstructed labor. Demographic characteristics and outcomes were compared between colonized and noncolonized participants using chi-squared or Fisher's exact test for categorical variables and Student's *t*-test or the Wilcoxon rank sum test for continuous variables. *P* values <0.05 were considered statistically significant. A multivariable logistic regression model was used to identify factors associated with vaginal potential pathogen colonization. Variables with a *P* value >0.05 were selected for inclusion in the model which included the following risk factors for vaginal colonization: age, distance of residence from hospital, marital status, educational status, referral status, number of vaginal exams prior to delivery, and parity. All analyses were performed using Stata software (version 12.0, StataCorp, College Station, TX).

### 2.8. Ethical Clearance

The study and enrollment procedures were approved by the Mbarara University of Science and Technology institutional research committee (08/10–14), Partners Healthcare (2014P002725/MGH), and the Uganda National Council of Science and Technology (HS/1729).

## 3. Results

### 3.1. Enrollment and Sample Collection

**Figure 1:**
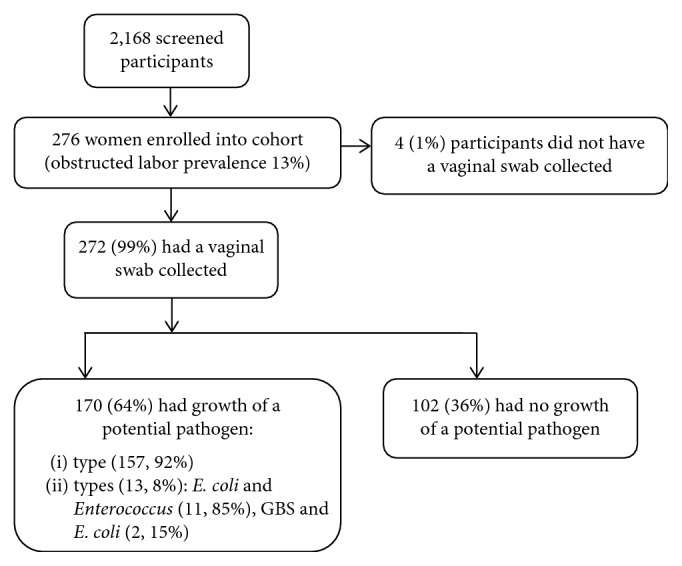
Flowchart of participant enrollment and sample collection.

Of 2,168 laboring women consecutively screened between April and October 2015, 276 (13%) women met criteria for obstructed labor (Figure 1). Of these, 272/276 (99%) had a vaginal swab collected and 170/276 (64%) swabs grew one or more potential pathogens. Of the 170 swabs tested, 157 (92%) grew one potential pathogen and 13 (8%) grew two potential pathogens (11 with both *E. coli* and *Enterococcus* and two with both GBS and *E. coli)*. *E. coli* was identified in 134 (79%) of positive vaginal swab cultures, *Enterococcus* in 22 (13%), and GBS in 14 (8.2%), including both samples that grew one potential pathogen and those that grew two pathogens. The overall proportion of *E. coli* colonization was 49%, *Enterococcus* was 8%, and GBS was 5%.

### 3.2. Participant Characteristics and Exposures by Colonization Status

There was no significant difference in demographic factors between women having vaginal colonization with a potential pathogen and those not colonized (Table 1), including age (*P*=0.97), residence in the same municipality as the hospital (*P*=0.95), marital status (*P*=0.09), and employment status (*P*=0.81). There were also no differences in obstetric factors including parity (*P*=0.71), gestational age (*P*=0.13), number of vaginal examinations (*P*=0.67), and antenatal care attendance (*P*=0.50). The duration of labor was significantly longer in women having vaginal colonization with GBS, *E. coli*, or *Enterococcus* compared with those not colonized (median 24 versus 13 hours, *P*=0.03; Table 1).

### 3.3. Maternal and Neonatal Clinical Outcomes

All women enrolled in the study underwent cesarean delivery. Maternal and neonatal outcomes did not differ between women vaginally colonized with a potential pathogen and those not colonized, including endometritis (8 (40%) versus 6 (55%), *P*=0.44), postpartum fever or hypothermia (20 (12%) versus 11 (11%), *P*=0.81), death (1 (1%) versus 0 (0), *P*=0.63), postpartum hemorrhage (1 (1%) versus 1 (1%), *P*=0.70), stillbirth (8 (5%) versus 3 (3%), *P*=0.50), or in-hospital neonatal death (1 (1%) versus 2 (2%), *P*=0.28) (Table 2).

### 3.4. Factors Associated with Colonization

In both univariable and multivariable logistic regression analyses, we controlled for potential confounders including duration of labor, HIV serostatus, age, and number of vaginal exams prior to delivery. After adjusting for potential confounders, number of hours in labor was the only significant independent risk factor for vaginal colonization by a potential pathogen (adjusted odds ratio (*a*OR) 1.02, 95% CI 1.00–1.03, *P*=0.04; Table 3).

### 3.5. Antimicrobial Susceptibility

Among GBS isolates, 5 (38%) were resistant to penicillin and among *E. coli* isolates, 85 (61%) were resistant to ampicillin, 5 (4%) to gentamicin, 6 (4%) to ciprofloxacin, and 7 (5%) to both ceftriaxone and cefepime (Table 4). No enterococci were resistant to either ampicillin or vancomycin.

## 4. Discussion

The prevalence of obstructed labor in our study was 13%, similar to what has been reported in other resource-limited settings including Ethiopia (11%) and southwestern Uganda (8%) [13, 14]. Contrary to our hypothesis, we found no significant difference in adverse maternal or neonatal outcomes between women with obstructed labor who had vaginal colonization with *E*. *coli*, GBS, or enterococci compared with those not colonized. Our primary outcome, postpartum endometritis, occurred in 8 (40%) in the colonized women and 6 (55%) among the noncolonized group (*P*=0.44).

Our primary finding of no difference in clinical outcomes between women colonized and those not colonized with a potential pathogen may reflect a number of factors. Since it is standard practice at MRRH for women in labor to have vaginal cleansing with chlorhexidine before any pelvic procedure is done, potential pathogens present in the vaginal canal may have been reduced or eliminated by chlorhexidine, preventing adverse outcomes in these women. Chlorhexidine has activity against vaginal bacteria, which causes puerperal infection, including GBS, *E. coli*, and enterococci [15]. The small overall number of adverse maternal outcomes (14 (5%) developed endometritis) and fetal outcomes (13 (5%) stillbirths) would have also contributed to the finding of no significant difference in the outcomes between the two groups. The small numbers also led our study to be underpowered, reducing our ability to detect a difference in outcomes between the two groups. Because prior studies have focused on infections caused by these potential pathogens [16–18] and not specifically on asymptomatic colonization, it is difficult to compare our finding of a lack of association between vaginal colonization and outcomes with prior studies. It is also possible that previous research demonstrating increased risk of poor outcomes among women with GBS, *E. coli*, and *Enterococcus* infections may reflect colonization with particularly virulent strains of these bacteria, and we did not measure pathogen virulence in our study. However, among women colonized with potentially pathogenic bacteria, we presume that virulent strains are more likely to be associated with clinical infection than less virulent colonizers and may account for differences in infectious outcomes between studies. It is also possible that the three pathogens selected for this study may not be the most important pathogens causing adverse pregnancy outcomes, or that a combination of pathogens or the total microbial load and environment of the vagina is a more important determinant of outcomes. These are potential areas for future research.

Our description of the microbiology and antimicrobial susceptibility of three important bacteria among a high-risk population of laboring women may be useful to determine empiric antibiotic treatment for women with obstructed labor who develop peripartum infections in resource-limited settings. We found that the proportion of *E. coli* vaginal colonization during obstructed labor was 49%, higher than 13–23% reported colonization in other populations of nonobstructed laboring women [19–22]. Higher *E. coli* colonization rates may reflect an increased number of vaginal exams during labor or increasing vaginal contamination by anorectal bacteria as a result of prolonged or obstructed labor. Because maternal vaginal bacterial colonization with *E. coli* is associated with early onset neonatal sepsis, postpartum endometritis, urinary tract infections, and chorioamnionitis [8, 23], *E. coli* antibiotic susceptibility patterns have important implications for empiric antibiotic selection. In resource-limited settings, common empiric antibiotics for neonatal sepsis, endometritis, urinary tract infection, and chorioamnionitis often include ampicillin, gentamicin, ceftriaxone, ciprofloxacin, or some combination of these first-line antibiotics. We found that 61% of *E. coli* vaginal isolates were resistant to ampicillin, similar to what has been described elsewhere [21, 24]. We found low-level resistance for *E. coli* to ceftriaxone, gentamicin, and ciprofloxacin, which is reassuring that one or more of these commonly used empiric antibiotic choices are likely to be effective against *E. coli* infections in this setting.

We found a low prevalence (5%) of vaginal GBS colonization in our study population, lower than estimates from other resource-limited settings, ranging from 8 to 31% [25–27], including a prior study at MRRH among pregnant nonlaboring women which demonstrated 29% combined vaginal/rectal GBS colonization [26]. A five percent (5%) prevalence of vaginal GBS colonization is surprisingly low for this setting, especially as all vaginal swabs were collected prior to cleaning the vulva with chlorhexidine and prior to giving prophylactic antibiotics for Cesarean delivery. The low GBS prevalence could be partially explained by antibiotic use prior to hospital arrival or vaginal cleaning at home prior to admission, though presumably this would have also affected women in the previous MRRH cohort that found a higher GBS prevalence. We collected samples from the vagina only, not the rectum, which may also partially explain our low GBS colonization prevalence. Lastly, women in our study were enrolled in advanced labor, which differs from prior studies done during 2nd and 3rd trimester, but not during labor. Current local practice does not include screening or treatment of vaginal GBS colonization during pregnancy. Compared with a prior study finding 0% resistance to penicillin among vaginal GBS isolates [28], we found 38% resistance of GBS to penicillin. It is difficult to interpret the significance of these results, given the small number of isolates in our study.

Lastly, we found 8% vaginal enterococcus colonization in our study, the same proportion as in another study in pregnant women [9]. All enterococci isolated in our study were susceptible to both ampicillin and vancomycin, suggesting that postpartum enterococcal infections would likely be well treated by commonly used empiric antibiotic regimens, which usually include ampicillin.

There are several strengths to our study, including its prospective design and collection of vaginal swabs from nearly all (99%) participants with obstructed labor. Some weaknesses of the study include incomplete chart documentation which could have affected the outcome information abstracted from charts. Though research assistants carefully examined participants' charts for these outcome informations, they were only able to abstract what was recorded by the clinical team. Information about perisurgical antibiotic receipt in the hospital charts was also incomplete. In addition, we followed women physically only until hospital discharge, completing the two-week and six-week follow-up questionnaires by phone. Infections developing after discharge would not have been captured in our results and could be an important source of postpartum morbidity among women with obstructed labor. Lastly, we only performed one exam at three days postpartum to diagnose endometritis among study participants. For this reason, women developing endometritis more than three days postpartum but prior to hospital discharge would also have been missed.

## 5. Conclusions

We found no difference in maternal and neonatal clinical outcomes among women with obstructed labor between those who had vaginal colonization with a potential pathogen and those not colonized. Increased duration of labor was associated with increased risk of vaginal colonization, and the microbiology of vaginal swabs was dominated by Gram-negative rods. Increasing availability of microbiology testing to inform appropriate antibiotic use and determine the composition of vaginal flora around the time of delivery in patients with obstructed labor, and strengthening antimicrobial stewardship programs should be prioritized.

## Figures and Tables

**Table 1:** Demographic characteristics and obstetric risk factors of obstructed labor participants, comparing women vaginally colonized with a potential pathogen to those not colonized.

Characteristic (*N*=272)	Colonized, *n*=170 (63%)	Not colonized, *n*=102 (37%)	*P* value
Age (years)			0.97
≤19	34 (21)	19 (21)	
20–34	117 (75)	68 (75)	
>34	6 (4)	4 (4)	
Residence in Mbarara municipality	37 (24)	20 (24)	0.95
Married	143 (91)	88 (97)	0.09
No formal employment	46 (29)	28 (31)	0.81
HIV-infected	10 (6)	9 (9)	0.36
Median household monthly income in UGx (USD)	150,000 (42)	100,000 (28)	0.10
Referred to MRRH from another health facility	62 (40)	33 (36)	0.57
Attended ≥4 ANC visits	107 (70)	65 (72)	0.50
Received malaria prophylaxis during pregnancy (or TMP/SMX prophylaxis if HIV-infected)	151 (96)	88 (97)	0.83
Reported syphilis or sexually transmitted infection during pregnancy	0 (0)	2 (2)	
Primiparous	94 (60)	50 (55)	
Gestational age at delivery			0.13
Preterm (<37 weeks)	6 (4)	7 (8)	
Term (37–42 weeks)	125 (85)	74 (87)	
Postterm (>42 weeks)	16 (11)	4 (5)	
Reported ≥5 vaginal exams during labor	11 (13)	22 (15)	0.67
Estimated duration of labor in hours, median (IQR)	24 (12–43)	13 (11–24)	0.03
Presurgical antibiotic prophylaxis prescribed	135 (87)	80 (88)	0.76

UGx: Uganda shilling; USD: United States dollar; MRRH: Mbarara Regional Referral Hospital; ANC: antenatal care; TMP/SMX: trimethoprim/sulfamethoxazole; IQR: interquartile range.

**Table 2:** Maternal and neonatal outcomes of obstructed labor participants, comparing women vaginally colonized with a potential pathogen to those not colonized.

Outcome	Colonized, *n*=170 (63%)	Not colonized, *n*=102 (37%)	*P* value
*Maternal*			
Endometritis	8 (40)	6 (55)	0.44
Postpartum fever (>38.0°C) or hypothermia (<36.0°C)	20 (12)	11 (11)	0.81
Maternal death	1 (1)	0 (0)	0.63
Days hospitalized, mean (SD)	3.4 (3)	3.5 (3)	0.83
Reoperation	1 (1)	0	0.45
Postpartum urinary tract infection	1 (5)	1 (10)	0.61
Postpartum hemorrhage	1 (1)	1 (1)	0.70
Readmission to MRRH within 6 weeks postpartum	2 (1)	2 (2)	0.60

*Neonatal*			
Live birth	156 (100)	91 (99)	0.19
Stillbirth	8 (5)	3 (3)	0.51
In-hospital neonatal death	1 (1)	2 (2)	0.28
1-minute Apgar <7	11 (12)	11 (7)	0.24
5-minute Apgar <7	0 (0)	3 (3)	0.05

SD: standard deviation; MRRH: Mbarara Regional Referral Hospital.

**Table 3:** Univariable and multivariable logistic regression analysis of factors associated with vaginal colonization by a bacterial pathogen.

Characteristic	Univariable		Multivariable	
	cOR (95% CI)	*P* value^*∗*^	aOR (95% CI)	*P* value^*∗*^
Number of hours in labor	1.02 (1.00–1.03)	0.03	1.02 (1.00–1.03)	0.04
Number of vaginal exams in labor	0.97 (0.87–1.08)	0.58	0.95 (0.84–1.07)	0.40
Multiparous	0.89 (0.60–1.32)	0.58	0.9 (0.41–8.95)	0.41
Formally employed	0.93 (0.53–1.64)	0.81	0.77 (0.40–1.09)	0.43
HIV-infected	0.65 (0.25–1.65)	0.36	0.88 (0.28–2.76)	0.83
Age (years)	0.99 (0.94–1.04)	0.59	1.0 (0.92–1.09)	0.99
Residence in Mbarara district	0.98 (0.52–1.83)	0.95	1.0 (0.51–2.00)	1.0

HIV: human immunodeficiency virus; CI: confidence intervals; OR: odds ratio; cOR: crude odds ratio; aOR: adjusted odds ratio. ^*∗*^Tests of association between cohort characteristics and the presence or absence of vaginal colonization were performed using univariable or multivariable logistic regression analysis.

**Table 4:** Proportion of potential vaginal pathogens testing resistant or intermediate susceptibility to selected antimicrobials.

*N*	Pen (*n*, %)	Amp (*n*, %)	Ctx (*n*, %)	Cef (*n*, %)	Gent (*n*, %)	Ery (*n*, %)	TMP/SMX (*n*, %)	Tet (*n*, %)	Van (*n*, %)	Cip (*n*, %)
GBS (*n*=14)	5/14 (38)	—	—	—	—	3/14 (21)	12/14 (92)	12/14 (92)	—	—
*E. coli* (*n*=139)	—	85/139 (61)	7/139 (5)	7/139 (5)	5/139 (4)	—	104/139 (75)	—	—	6/136 (4)
*Enterococcus* (*n*=56)	—	0/29 (0)	—	—	0/56 (0)	—	—	—	0/49 (0)	—

Pen: penicillin; Amp: ampicillin; Ctx: ceftriaxone; Cef: cefepime; Gent: gentamicin; Ery: erythromycin; TMP/SMX: trimethoprim/sulfamethoxazole; Tet: tetracycline; Van: vancomycin; Cip: ciprofloxacin.

## Data Availability

The STATA data used to support the findings of this study are available from the corresponding author upon request.
